# Retroperitoneal dendritic cell sarcoma: A case report

**DOI:** 10.1097/MD.0000000000024459

**Published:** 2021-03-05

**Authors:** Chuanhong Wang, Pinggui Lei, Yong Wan, Ping Fu, Bing Fan, Jiaqi Liu, Fangfang Hu, Rongchun Xu

**Affiliations:** a Department of Radiology; b Department of Pathology; c Department of General Surgery, Jiangxi Provincial People's Hospital Affiliated to Nanchang University, Nanchang; d Department of Radiology, the Affiliated Hospital of Guizhou Medical University, Guiyang, China.

**Keywords:** interdigitating dendritic cell sarcoma, magnetic resonance imaging, tomography, X-ray computed

## Abstract

**Ratioanle:**

Interdigitating dendritic cell sarcoma (IDCS) is a rare sarcoma that originates from interdigitating dendritic cells in lymphoid tissue, the imaging characteristics of which are poorly defined. Pathological examination can identify the tumor, but reports on the imaging characteristics of IDCS are limited.

**Patient concerns:**

Here, we report a case of IDCS in a 48-year-old female involving the retroperitoneal area. The patient had a lumbar mass on her right lower back for 4 years, and which started increasing in size 1 year before.

**Diagnoses:**

An irregular soft tissue mass (10.1cm × 8.5 cm in size) in the right lower back of retroperitoneum was detected by CT examination with unclear borders, uneven density, and necrosis. The solid components of the mass were significantly enhanced on postcontrast imaging. The soft tissue was irregular and uneven. Cystic solid masses were observed on MRI examination in the right retroperitoneum, lateral abdominal wall, waist, and back. Necrosis, hemorrhage, and cystic transformation were observed inside the lesion. The cyst wall, separation, and wall nodules were significantly enhanced on the postcontrast image. No distant metastasis was observed. Postoperative pathology confirmed the diagnosis of IDCS.

**Interventions:**

The patient underwent surgical resection. The resected margin was positive, and the patient received adjuvant radiotherapy 2 months after the surgery.

**Outcomes:**

Twelve months after radiotherapy, the patient's chest CT showed multiple metastases in both lungs. The patient was started on combination chemotherapy of doxorubicin and ifosfamide, and the follow-up is still ongoing.

**Lessons:**

Imaging provides a unique advantage to determine the extent of the IDCS, the invasion of adjacent tissues, and the presence or absence of distant metastases.

## 1 Introduction

Interdigitating dendritic cell sarcoma (IDCS) is a rare sarcoma that originates from interdigitating dendritic cells in lymphoid tissues.^[1]^ IDCS is most common in the lymph nodes, and typically manifests as a painless enlargement of the lymph nodes, mostly in cervical lymph nodes, but also as primary extranodal lesions.^[2]^ The etiology and pathogenesis of IDSC are unknown.^[3]^ Pathological examination can identify other tumors, but reports on the imaging characteristics of IDCS are limited. In this study, we report a single case of huge retroperitoneal IDCS in a 48-year-old female and describe the imaging characteristics. This study was approved by ethics committee of the hospital. The patient signed informed consent.

## 2 Case presentation

**Figure 1:**
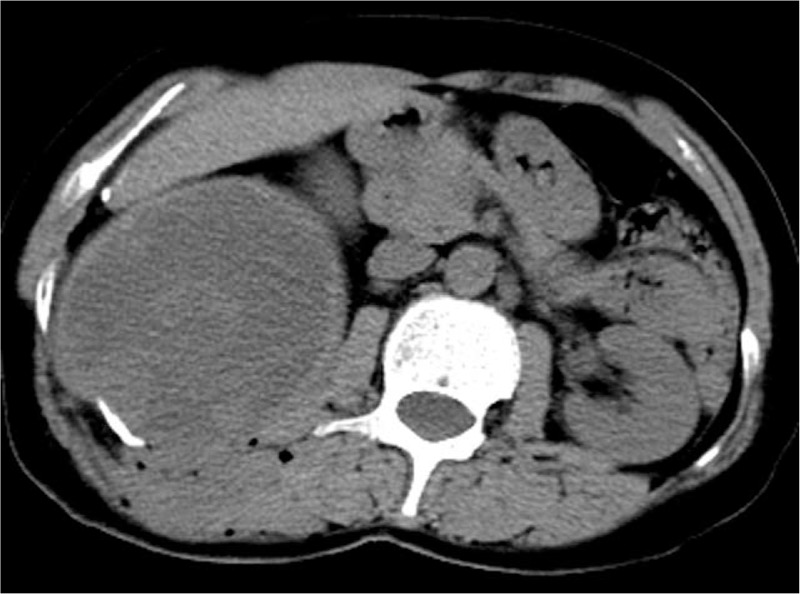
Plain CT scans of the right lower back muscle soft tissue and retroperitoneal low-density cystic mass, the soft tissue nodular wall can be almost seen.

**Figure 2:**
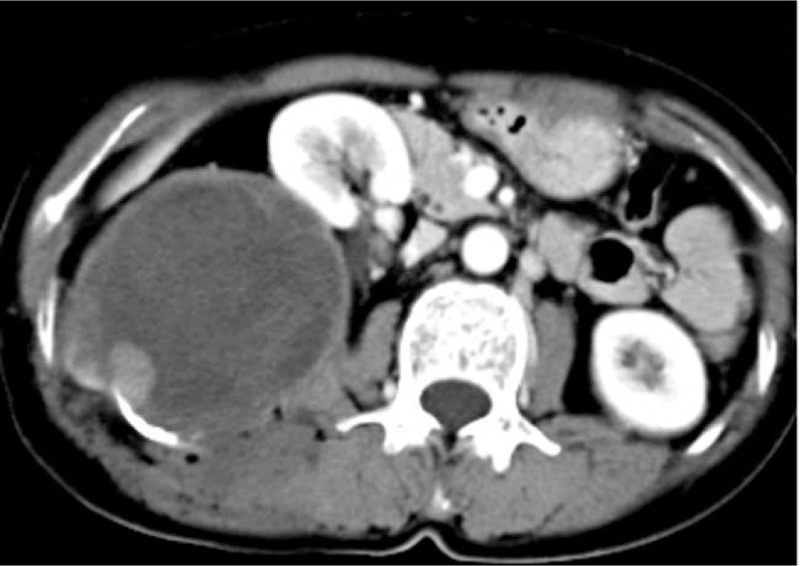
Solid components of CT-enhanced masses were unevenly enhanced and some cyst walls were nodularly enhanced, and the cystic lesion showed no enhancement.

**Figure 3:**
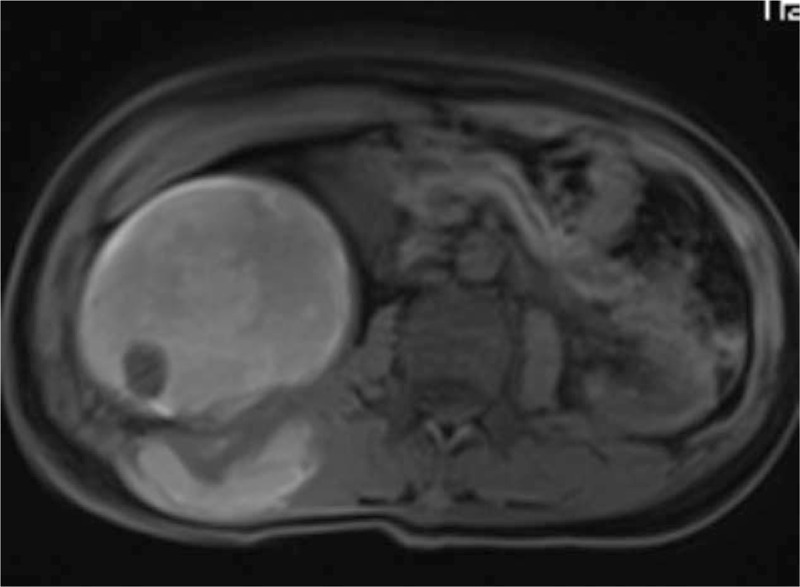
MRI: The nodular of wall showed equal or slightly lower signals. However, most of the cystic area revealed equal or slightly high signals.

**Figure 4:**
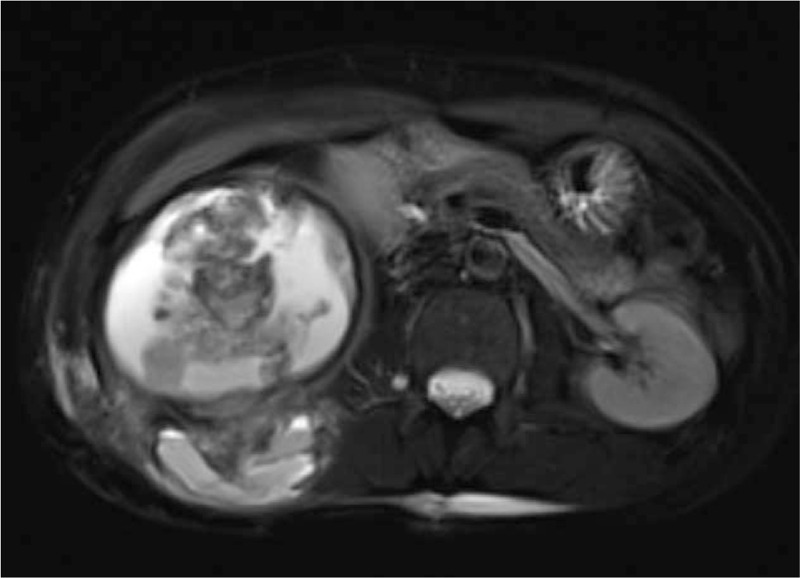
MRI: T2WI mass showed high and low mixed signals, but mainly high signals in the cystic area.

**Figure 5:**
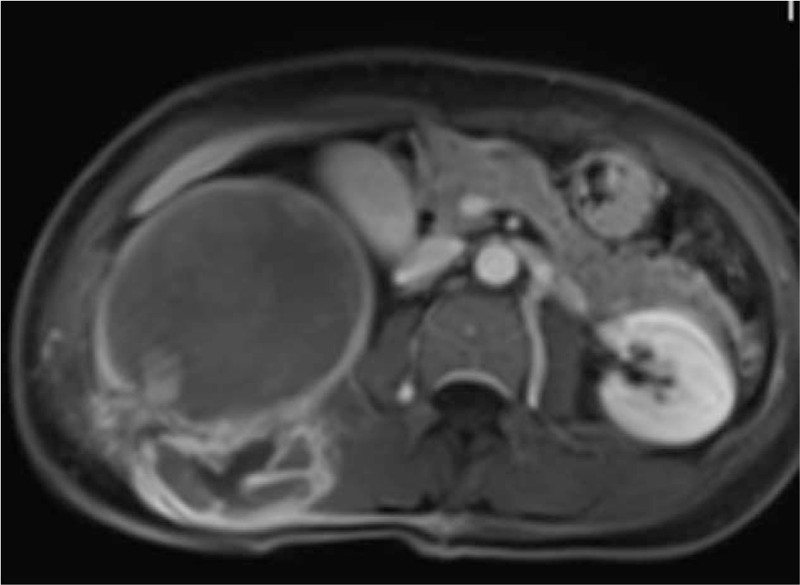
Cyst walls, partition, and mural nodules of the mass were significantly enhanced in the postcontrast T1WI with fat suppression.

The patient was a female aged 48 years old, who had a lumbar mass on her right lower back for 4 years, and was admitted to hospital more than a year after noticing the enlargement of the mass. The mass on the right lower back was unintentionally discovered 4 years earlier and was approximately 2 × 2 cm in size. The patient did not experience any discomfort such as chills, fever, or local pain. The mass began to enlarge 1 year earlier without discomfort. The past surgical history included total hysterectomy in 2013. On examination, a cystic mass of about 5 × 5 cm was observed in the right lower back, with clear borders, smooth surface, hard on palpation, no pressure pain, and no mobility. Laboratory examinations showed decreased hemoglobin levels of 102 g/L (normal value: 130–175 g/L). Liver function, renal function, AFP, CEA, and CA199 were normal. Color Doppler ultrasonography showed mixed echoes on the right lower back. Plain CT scans showed irregular low-density cystic masses on the right retroperitoneum and on the soft muscles of the back. The size was about 10.1 × 8.5 cm. The borders were unclear and the density was irregular. The solid region of enhanced scans showed uneven strengthening, with the mural wall showing nodular strengthening (Figs. 1 and 2). Plain MRI scans showed mixed-signal cystic solid masses in the right retroperitoneum and lower back, with necrotic bleeding, and cystic changes in the masses. T1WI showed equal or slightly lower signals, with T2WI showing high signals. The cyst wall, partition, and mural nodules were significantly strengthened, and the right kidney and liver moved forward under pressure. The boundaries with subcutaneous and surrounding muscles were unclear (Figs. 3–5).

**Figure 6:**
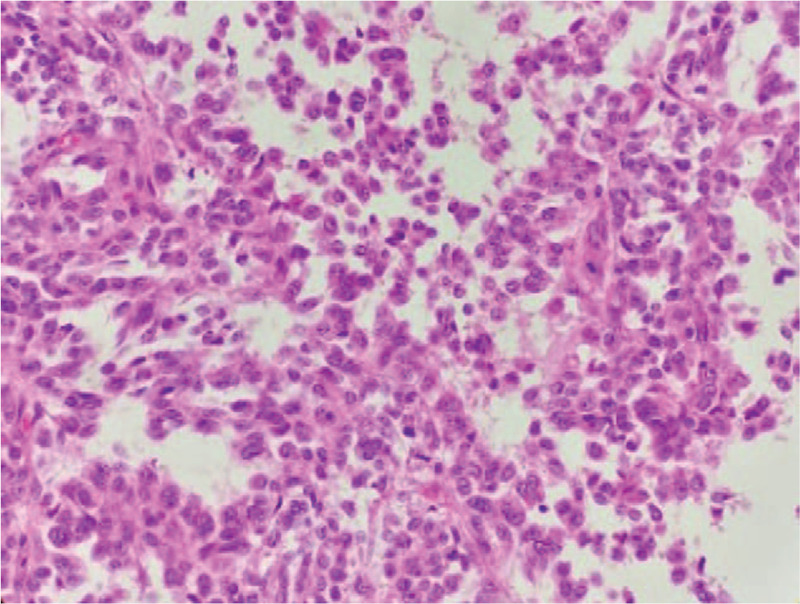
Tumor cells were round or oval, the chromatin was coarse-grained, and mitotic cells were visible (HE staining, ×400).

**Figure 7:**
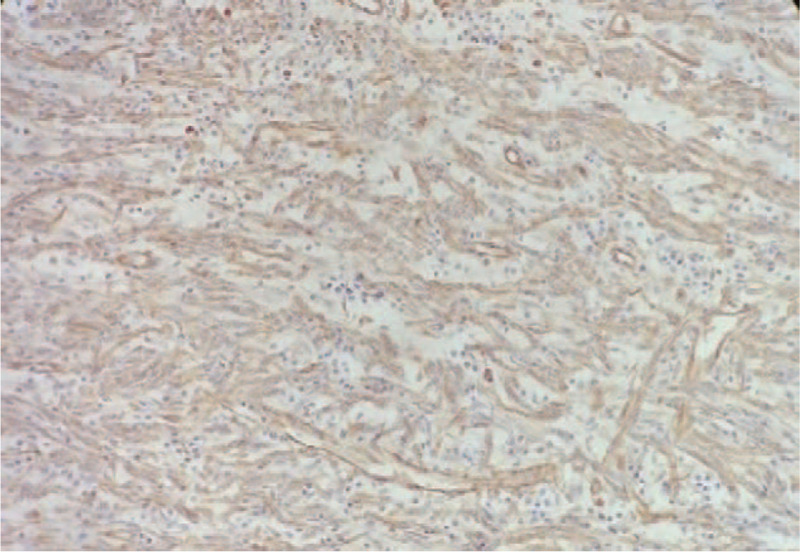
Tumor cells are round or oval under high magnification, the cytoplasm is transparent, the nucleus is round or oval, the chromatin is coarse-grained, the nucleolus is obvious, and the mitotic figures are present. Immunohistochemistry suggested positive for vimentin, CD68, and CD163.

**Figure 8:**
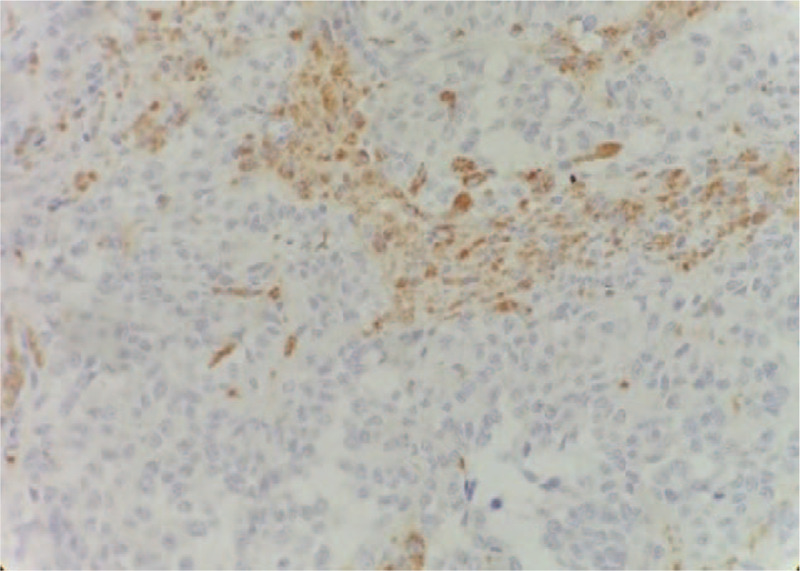
Tumor cells are round or oval under high magnification, the cytoplasm is transparent, the nucleus is round or oval, the chromatin is coarse-grained, the nucleolus is obvious, and the mitotic figures are present. Immunohistochemistry suggested positive for vimentin, CD68, and CD163.

**Figure 9:**
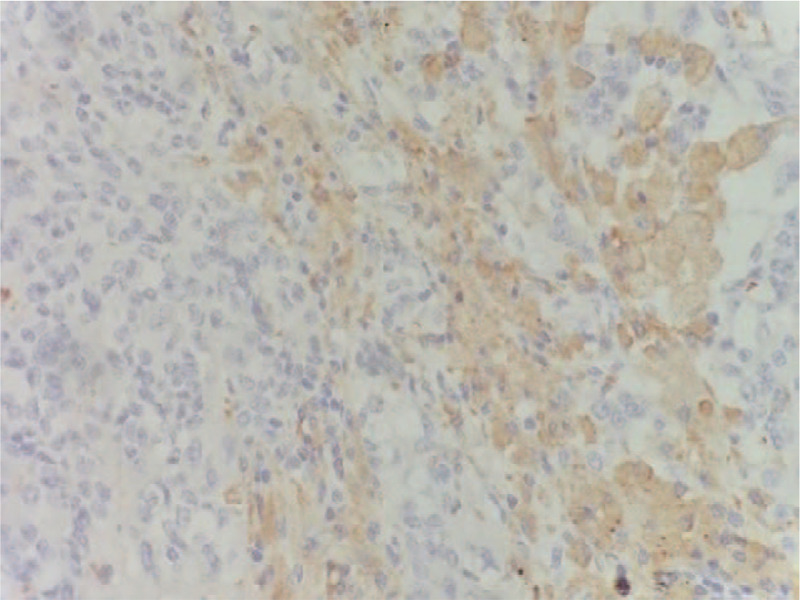
Tumor cells are round or oval under high magnification, the cytoplasm is transparent, the nucleus is round or oval, the chromatin is coarse-grained, the nucleolus is obvious, and the mitotic figures are present. Immunohistochemistry suggested positive for vimentin, CD68, and CD163.

Biopsy pathology prompted the diagnosis of a solid-pseudopapillary tumor, and surgical treatment was provided. Intraoperatively, a giant cystic solid tumor was observed behind the right retroperitoneum, located extremely behind the right kidney below the liver. The tumor pushed the right kidney forward and medially, and the medial side was adjacent to the inferior vena cava, the right adrenal gland, and the right diaphragm. The dorsal base of the tumor invaded the lower back muscles. The tumor, right adrenal gland, part of the diaphragm and lower back muscles were removed. The tumor had a positive resected margin, and the tumor invaded the fascia. The size of the specimen was about 10 × 8 × 5 cm. Histopathology showed tumor cells arranged in a papillary shape under a light microscopy. The cells were round or oval, the nuclei were round, and the chromatin was coarse-grained. The nucleoli were obvious and mitotic cells were abundant (Fig. 6). Immunohistochemistry findings: Vimentin(+), S-100(+), CD68(+), CD163(+), CD1a(−), CD21(−), β-catenin(cytoplasm+), ER(−), PR(weak+), Fli(−), CD99(−), Catenin-b(−), CD10(−), CK(AE1/AE3)( −), cyclin D1(−), Galectin-3(−), EMA (weak +), Ki-67 (+, about 10%), the cell atypia was obvious. Electron microscopy was not performed due to limited conditions. IDCS was diagnosed based on cell morphology and immunohistochemical examination (Figs. 7–9). There was no tumor involvement in the right adrenal gland. The patient received adjuvant radiotherapy for 2 months after the surgery. The radiotherapy dose was DT 50GY/25F. After radiotherapy, the patient's white blood cells were reduced to 2.13 × 109/L. No obvious tumor recurrence or metastatic signs were observed in the abdomen during 4 months of follow-up. Chest CT showed multiple metastases in both lungs 12 months after radiotherapy. He was given doxorubicin + ifosfamide combined chemotherapy and is still under follow-up.

## 3 Discussion

IDCS is a rare sarcoma that originates from interdigitating dendritic cells. To-date, approximately 100 cases have been reported in the literature. The etiology and pathogenesis of IDCS remain unclear. Tumorigenesis is frequent in adults, and occurs most frequently in males (male to female ratio: 1.65:1).^[4]^ IDCS mainly occurs in the lymph nodes, but can occur in the liver, spleen, skin, lung, small intestine, nasopharynx, mesentery, testis, tonsils, bone marrow, chest wall, bladder, and breasts.^[5]^ The clinical manifestations are non-specific, ranging from painless lymphadenectasis to symptoms related to the compression or impaired function of the affected tissues and organs. Systemic symptoms are rare. Tumor lumps generally display a leaf-like appearance, and the cut surface is moderately grayish. The tumors are frequently accompanied by necrosis, bleeding, and invasion toward surrounding tissues. The diagnosis of IDCS depends on clinical, histopathology, immunophenotype, electron microscopy, and histopathology.^[6]^ Under low power microscope, the tumors are distributed in sheets, and the tumor cells are arranged in swirls, bundles and sheets. The tumor cells have obvious atypia, with unclear cell boundaries. The cells are fusiform, fat fusiform, oval, with histiocyte-like morphology, fine chromatin, clear nuclear membrane, mitotic figures, and some nuclei are finger-shaped. The nucleus lobes are visible, and there are generally no tumor giant cells. Most of them have no necrosis. Reactive inflammatory cell infiltration is seen in the interstitium.^[7]^ IDCS has histiocyte/dendritic cell characteristics. Generally, it is positive for S-100 protein, CD68, Vimentin, and Fascin, but it is not specific. The positive rate of S-100 protein is higher. Compared with follicular dendritic cells and Langerhans cells of the same family, tumor cells are negative for CD21, CD23, CD35, D2–40, CD207, CD1a, and CK. Secondly, tumor cells do not express melanin (HMB45, Melan A, PNL2, etc.), Myogenic markers (desmin, MyoD1, Myogenin).^[8]^ Histological and immunohistochemical examination of this case was typical and met the diagnostic criteria of IDCS.

Reports regarding IDCS imaging are limited. Zhu reported a case of IDCS originating from the sigmoid colon mesentery, showing a solid mass with a rich blood supply, with foci that were significantly enhanced by CT-enhanced scanning. No clear cystic components were found.^[9]^ In this case, the imaging findings showed a giant cystic solid mass on the right retroperitoneum and lower back, with unclear borders. The mural wall, partition, and wall nodules around the enhancement were strengthened, and most of the foci were cystic areas lacking strengthening. The foci were large and liquefaction necrosis was caused by the poor blood supply in the center of the foci. Due to the rare incidence of IDCS, it is challenging to diagnose and preoperative biopsy in our case was incorrectly diagnosed as solid pseudopapillomas. Although CT and MRI examinations cannot be used as confirmation, they have unique advantages in determining the scope of the focus, the invasion of adjacent tissues, and distant metastases. This can reflect the biological behavior of the tumor and guide the formulation of surgical treatment plan. The diagnosis should be based on a comprehensive analysis of the histology and immunohistochemistry of the tumor, and if necessary, electron microscopy assessments.

To-date, there is no standard treatment plan for patients with IDCS. For localized foci, surgical resection or adjuvant radiotherapy is preferred, and IDCS with multiple systemic metastases usually displays a poor prognosis.^[10]^ Based on the Cox regression model, surgical resection is the only treatment associated with improved survival. The 1-year mortality rates in resected and non-resected disease were 17.8% and 63.2%, respectively (*P* < .01). The median overall survival (OS) and median progression-free survival (PFS) were 12 and 6 months, respectively.^[4]^ Recurrence after surgery is common. Cellular atypia and mitosis show no association with survival.^[5]^

In summary, IDCS is a rare tumor that manifests as a vascular-rich tumor, with larger tumors showing liquefaction necrosis. Imaging has the unique advantage of determining the extent of the focus, the invasion towards adjacent tissues, and the presence or absence of distant metastases.

## Author contributions

WCH carried out the data collection, literature review and drafting of the manuscript. WY and FP contributed to the drafting of the manuscript and aided in the literature review. LJQ and HFF participated in the data collection and the drafting of the manuscript. PGL, BF and XRC help to design the work and revised the final version of the manuscript. All authors read and approved the final manuscript.

**Conceptualization:** Chuanhong Wang, Bing Fan, Rongchun Xu.

**Data curation:** Chuanhong Wang, Ping Fu, Bing Fan, Jiaqi Liu, Fangfang Hu, Rongchun Xu.

**Formal analysis:** Chuanhong Wang, Pinggui Lei, Yong Wan, Ping Fu, Bing Fan, Fangfang Hu, Rongchun Xu.

**Funding acquisition:** Pinggui Lei, Bing Fan, Rongchun Xu.

**Investigation:** Chuanhong Wang, Pinggui Lei, Bing Fan, Jiaqi Liu, Rongchun Xu.

**Methodology:** Pinggui Lei, Yong Wan, Bing Fan, Jiaqi Liu, Fangfang Hu, Rongchun Xu.

**Project administration:** Yong Wan, Bing Fan, Rongchun Xu.

**Resources:** Chuanhong Wang, Ping Fu, Bing Fan.

**Software:** Rongchun Xu.

**Supervision:** Pinggui Lei, Yong Wan, Bing Fan.

**Validation:** Yong Wan, Bing Fan.

**Visualization:** Ping Fu, Bing Fan.

**Writing – original draft:** Chuanhong Wang, Ping Fu.

**Writing – review & editing:** Bing Fan, Rongchun Xu.
